# Global Geometric Affinity for Revealing High Fidelity Protein Interaction Network

**DOI:** 10.1371/journal.pone.0019349

**Published:** 2011-05-03

**Authors:** Yi Fang, William Benjamin, Mengtian Sun, Karthik Ramani

**Affiliations:** 1 School of Mechanical Engineering, Purdue University, West Lafayette, Indiana, United States of America; 2 School of Electrical and Computer Engineering, Purdue University, West Lafayette, Indiana, United States of America; Koç University, Turkey

## Abstract

Protein-protein interaction (PPI) network analysis presents an essential role in understanding the functional relationship among proteins in a living biological system. Despite the success of current approaches for understanding the PPI network, the large fraction of missing and spurious PPIs and a low coverage of complete PPI network are the sources of major concern. In this paper, based on the diffusion process, we propose a new concept of global geometric affinity and an accompanying computational scheme to filter the uncertain PPIs, namely, reduce the spurious PPIs and recover the missing PPIs in the network. The main concept defines a diffusion process in which all proteins simultaneously participate to define a similarity metric (global geometric affinity (GGA)) to robustly reflect the internal connectivity among proteins. The robustness of the GGA is attributed to propagating the local connectivity to a global representation of similarity among proteins in a diffusion process. The propagation process is extremely fast as only simple matrix products are required in this computation process and thus our method is geared toward applications in high-throughput PPI networks. Furthermore, we proposed two new approaches that determine the optimal geometric scale of the PPI network and the optimal threshold for assigning the PPI from the GGA matrix. Our approach is tested with three protein-protein interaction networks and performs well with significant random noises of deletions and insertions in true PPIs. Our approach has the potential to benefit biological experiments, to better characterize network data sets, and to drive new discoveries.

## Introduction

Current development in high-throughput measurement techniques such as tandem affinity purification, two-hybrid assays, and mass spectrometry have resulted in vast amounts of pertinent elements and the biological networks of their interactions [1]–[9]. The wealth of observed data has provided more opportunities and challenges in the exploration of protein functions and regulations in various organisms. However, the reliability of the observed PPIs is a major source of concern as the data inherently has very high rates of false measured interactions, sometimes up to 50% [10], [11], and a low coverage of the complete PPI network. Therefore, the computational approaches have been receiving an increasing attention towards assisting PPI network analysis [1], [12]–[14]. The general approach behind the computational methods combines the extra information (evidences) to re-evaluate the quality of the observed network. The assumption is that the observed PPI network itself is a realization of an underlying probabilistic model [1], [15]. If the probability 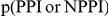 (NPPI denotes protein-protein non-interaction henceforth) is the probability of the observed PPIs and NPPIs in the network, statistically, the computational approaches seek for the evidences to maximize the posterior probability 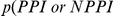
| 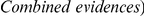. Therefore, the strategy to integrate the evidences of the observed PPI network plays a critical role in the improvement of the fidelity of protein-protein interaction. According to the different types of the methods for combining the evidences, we can categorize the current efforts into two aspects discussed below.

As measurements cover different aspects of a biological systems with different characteristics of the PPI network [16], the first type of approaches of combining evidences integrate the multiple data sources, such as gene expression arrays, proteomics, and chromatin immunoprecipitation on chip assays [17], [18], to evaluate the protein-protein interactions and its network, and then to improve the accuracy, coverage and robustness of PPIs. The integration methods typically include Bayesian approaches, decision tress, support vector machines and neural networks [12], [19]–[21]. Those methods have proven to be particularly effective and yielded a clearer biological view of the system than single source based methods. However, the high rates of uncertain PPIs pose a challenge to integrate different resources, and the experimental measurements are sometimes extremely labor intensive and expensive. The geometric features of the PPI network have recently proven to provide new insights into PPI network [1], [22]–[30]. The second type of approaches of combining evidences mines the geometric characteristics behind the PPI network to evaluate the quality of PPIs. The common hypothesis for these methods is that the existence of the geometric topology structure for PPI networks which is crucial in determining the PPIs in the network [22]. Those approaches are promising as they only require the input from the PPI network topology. Although it is a good initial step, there are still open research questions to answer in order to fully understand the PPI network based on its geometric features. The methods use the direct connections to evaluate the quality of an interaction pair [24], [25], [28], [29]. Those methods are basically similar to the method in [28]. If they have many common neighbors, a pair of nodes is likely to be connected in the network. The methods rely on the degrees of individual proteins without considering the entire topology of the network. This kind of methods are particularly effective however they are sensitive to noise as only local individual neighbors are considered in prediction and not sufficient to evaluate the global relation among nodes [26], [29]. The improved techniques in [24], [29] firstly cluster PPIs in the network in order to form sub-groups and then analyze the direct connections in the local cliques. However the methods are still strongly relying on the direct interactions. The approaches, which are able to exploit the entire topology based on using indirect neighbors in a network, are more promising [22], [26], [27] in revealing the true relationship among nodes in network. For example, a robust protein function prediction based on the indirect neighbors has been presented in the [27]. The state of the art approach uses a spectral decomposition, multidimensional scaling (MDS), to exploit the indirect neighbors in the network [1]. Their method firstly embedded the proteins in a high-dimensional space by the eigen-decomposition of the distance matrix, compute the Euclidean distance in the feature space, and then assign a PPI when such distance is smaller than a given threshold. A pair of proteins will be assigned an interaction (PPI) if they are close to each other in an embedded space whereas the NPPI corresponds to the pair of proteins that are distant from each other [1]. The performance of this method would be limited by the following drawbacks: the determination of the dimensionality used for embedding and the expensive computation cost due to the eigen-decomposition for large sized sparse or singular matrix. For the first issue, in [1] authors claim that the dimensionality might be related to the bio-chemical meaning but leave the strategy of determining optimal dimensionality in their future work. For the second issue, the current geometric methods unavoidably lead to a problem of the eigen-decomposition for a huge PPI matrix, which gives rise to computational difficulty in the real applications. In addition, the existing distance metric used to capture the geometric relationship is not robust to noise, for example, the geodesic distance in [1] is sensitive to short-circuit noise (turning the NPPI to false positive PPI). Therefore, the new defined metric, based on the embedding of geodesic distance in a Euclidean space by MDS, is unavoidably sensitive to short-circuit noise.

Similar to the method in [1], our method exploits the entire topology of the network based on spectral analysis. We proposed a novel computational framework based on a generalized diffusion process [31]–[33]. However, there are two main differences between those two methods. First, we do not compute the explicit representation for each protein in the feature space but the global implicit geometric affinity (GGA) by taking powers of a weight matrix about protein-protein relationship. Second, the GGA is revealed by the integration of local structure to a global geometric structure in a consistent manner, thus it is able to integrate the local limited sets of protein pairs to a global picture. Therefore, the new defined metric, based on the embedding of geometric in a Euclidean space, is more robust to short-circuit noise. Furthermore, our method addresses four challenging aspects: the determination of the optimal dimensionality, the limitations due to a low interaction coverage, the challenges of high rates of false positives and negatives, and the computational difficulty of eigen-decomposition for the large size of sparse and singular PPI network graph.

In addition, we are aware of the relationship between the GGA-method and Markov clustering (MCL) [28]. These two methods have the common in using random walk as theoretical basis. The differences lie in the following two aspects: 1) the application is different. MCL is proposed to cluster the network into small sub-networks. GGA is proposed to improve the fidelity of PPI network, 2) algorithmic difference in GGA and MCL. The MCL algorithm uses both the expansion and inflation operators iteratively to find out a steady state where the each value in a single column of the resulting matrix remains the constant. This convergence has not been proven yet. In other words, the MCL algorithm attempts to find an equilibrium state using the expansion and inflation operator on the transition matrix iteratively [28]. Our GGA algorithm uses the expansion operator progressively but attempts to realize the optimal scale of revealing the intrinsic relationship among proteins. The optimal scale for geometric structure of PPI network is determined by the probability based algorithm. The revealed scale does not necessarily indicate the equilibrium state but the state where the optimal intrinsic geometric structure is revealed.

**Figure 1:**
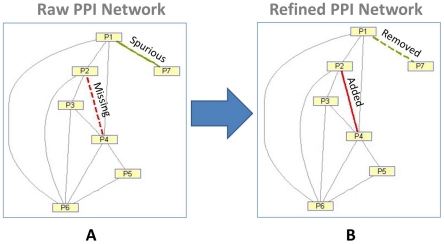
The illustration of the diffusion process based inference. (A) displays a spurious PPI (in green color solid line) and a missing PPI (in red color dash line). (B) shows that the spurious PPI (in green color dash link) is removed and the missing PPI (in red color solid line) is recovered in the network.

In **Figure 1**, we illustrate the general idea behind our method for removing the spurious PPI and recovering the missing PPI. In **Figure 1(A)**, we find that there is only a single and isolated path between P7 and P1 in the whole network, which is potentially the spurious protein-protein interaction. Meanwhile, we find that there are many paths bridging P2 with P4 in the network even when there is no direct link between the two. This type of pair of proteins is a potential PPI and missed by the observation in the experiment. In **Figure 1(B)**, we find that the spurious PPI is removed from the network and missing PPI is recovered.

## Methods

### Global geometric distance and affinity (GGD and GGA)

The computation of a global geometric metric from the local metric by geometric embedding operators has been recently established in machine learning research field [31], [33]–[38]. Two different ways for calculating the global geometric metric are diffusion based metric and geodesic distance based metric (i.e. shortest path). The diffusion based metric, defined in a diffusion propagation process, is usually more appropriate and robustly captures the global geometry of the original manifold. Its strength is derived from its ability to account for all “evidences” rather than the shortest path (a single evidence) relating one point to another one [31], [39]. For example, the authors in [39] found that diffusion metric based functional distances among protein domains closely correlate with sequence alignment, structural proximity and phylogenetic similarity. However, the geodesic metric based on the shortest path shows no significant correlation with either homology or phylogenetic similarity. Therefore, the diffusion metric is adopted in our method to evaluate the relationships among the proteins within a network. We will introduce a diffusion-based global geometric distance (GGD) and then develop our new global geometric affinity (GGA) in the following two sections.

#### Global geometric distance based diffusion metric (GGD)

**Figure 2:**
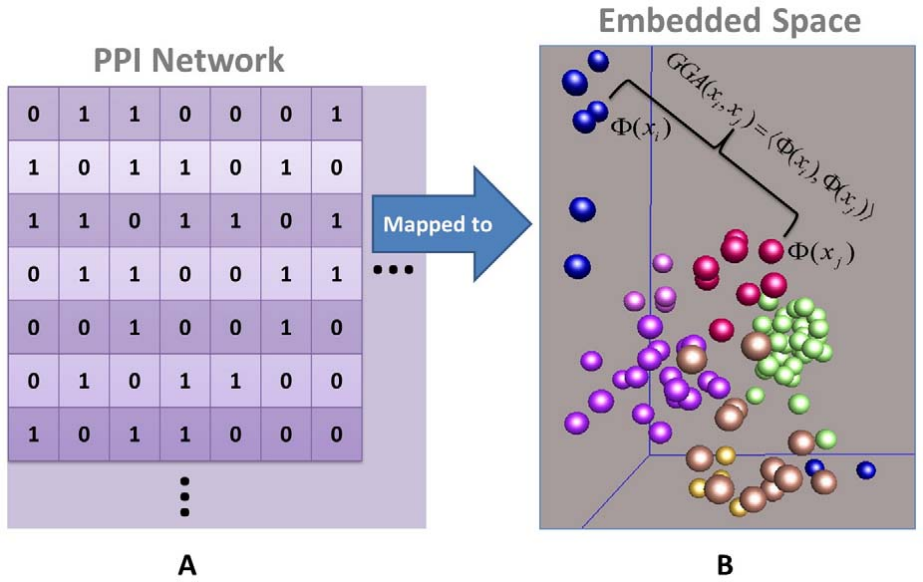
Geometric Representation of PPI network. (A) displays a PPI network. 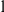 denotes the PPI and 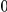 denotes NPPI. 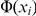 denotes the high dimensional feature vector of the point 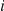 and 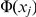 denotes the high dimensional feature vector of the point 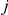 (a blue point and a red point). The 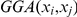 denotes the global geometric affinity between the point 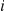 and point 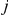.

The diffusion metric revealed in a diffusion propagation process reflects the intrinsic and geometric relationship among data points in the embedded diffusion space. In practice, the diffusion metric is in a form of geometric distance (dissimilarity), called diffusion distance, which is represented by the Euclidean distance in the embedded space [1], [31], [33]. In **Figure 2**, the PPI network (**Figure 2(A)**) is mapped to an embedded space (**Figure 2(B)**). In **Figure 2(B)**, 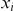 denotes the point 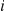 in the original space, the mapping operator 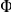 denotes the function that maps points in original space to embedded space, the 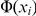 denotes the high dimensional vector in the embedded feature point. The diffusion distance 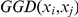 is formalized by the Equation 1 below:
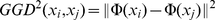
(1)


As the mapping function 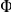 is usually unknown, the computation of the diffusion distance is usually calculated through the eigen-decomposition of the diffusion kernel [1], [31], [35], [36]. The distance is then approximated by the few leading eigenvectors corresponding to the few larger eigenvalue. The GGD is then formalized by the Equation 2 below:
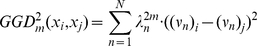
(2)Where 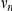 and 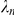 are the 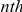 eigenvector and eigenvalue of the diffusion kernel respectively. The 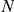 is the number of eigenvectors used for characterizing the embedding points (the dimensionality of the embedded space). The 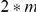 is the propagation steps. This computational scheme explicitly finds the approximated coordinates for the embedded points and computes the Euclidean distance among them, which, for example, is implemented by diffusion maps, ISOMAP and LLE [33], [35], [36]. However, as we discussed above, this typical computational scheme for global metric has drawbacks when applied to PPI network analysis such as the determination of optimal dimensionality and the computational difficulty due to the large size and singular matrix.

#### Global geometric affinity based on diffusion metric (GGA)

We introduce a new definition of geometric metric, called global geometric affinity (GGA), to overcome the weakness of current geometric based methods for PPI network. Different from the GGD, the GGA is in form of affinity (similarity), which is computed by the dot product of a pair of high-dimensional vectors (correlation coefficient). The 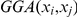 is formalized by the Equation 3 below:
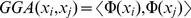
(3)Where the 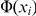 and 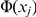 denote the high dimensional vector for point 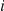 and point 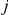 in the feature space respectively. The definition of GGA is the same as the definition of the kernel function by defining the dot product of feature vectors in the embedded space. In this paper, the GGA is defined based on a diffusion process by which the local affinity (similarity in original space) is integrated to a global affinity in a systematical manner. Under a generalized definition, any kernel function can be defined as diffusion type kernel only if it can map the local metric to a global picture. In [39], there is a complete review for different types of diffusion kernel definitions. In our paper, the GGA is defined by adapting the diffusion kernel in [31], [33] to PPI network. PPI network data is given in a graph format where nodes in graph correspond to proteins and two nodes are linked by an edge if the corresponding proteins interact with each other. Suppose we denote the PPI network as a graph 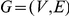, where 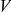 is the set of notes (proteins) and 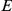 is a set of edges (PPIs), the adjacency matrix 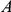 of a graph is a 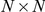 matrix in which the entry 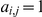 if there is an edge from node 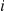 to node 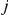 and is 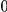 if there is no edge from node 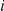 and node 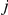. In our work, we can consider the adjacency matrix 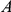 of PPI network a locally defined weighted matrix where weight is 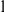 if two proteins interact and 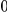 otherwise. The algorithm for the computation of GGA matrix (an 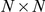 matrix whose entries are the GGA values for all protein pairs) defined as follows:

1. Initialization of the weight matrix:
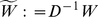
(4)And the symmetrized version of 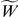 matrix is:
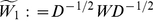
(5)Where 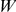 is the adjacency matrix 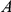 and 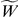 is the normalized weight matrix (also called local transition probability matrix), 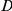 is a diagonal matrix and is defined by 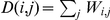 and 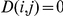 if 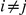, and 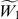 is the symmetrized version of 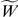. This normalization process described in [32], [39], however, is only optional in our application because PPI networks are often not well connected and very sparse, which gives trouble in the normalization process.
2. Propagation process:
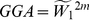
(6)Following Coifman et al. [32], the propagation process is implemented by taking powers of the matrix. Where 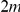 denotes the propagation steps and Coifman and Lafon [32] have suggested using even powers of 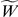.

The GGA reflects the internal affinity (geometric similarity) between node 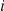 to 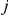 after 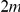 steps propagation as it considers all of possible paths between two nodes simultaneously via a diffusion process.

### Determination of optimal propagation step

**Figure 3:**
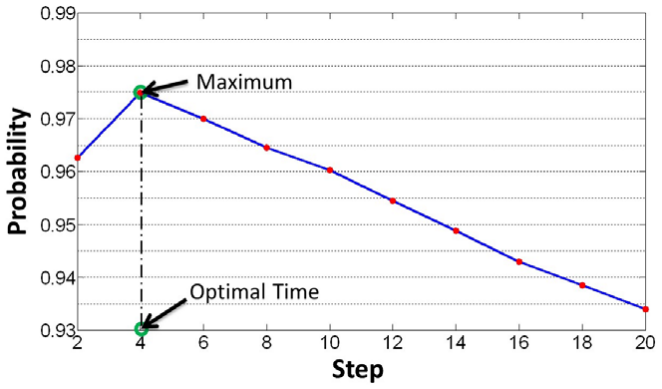
Determination of optimal propagation step. This figure illustrates the process of determination of the optimal step. The horizontal axis denotes the propagation step, and the vertical axis denotes the AUC based probability value. Based on the observation, probability value increases initially and monotonically decreases after obtaining its maximum at step around 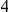.

The parameter, 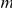 in Equation 6, determines different level of details of the internal relationship among proteins in network. To assess the optimal geometric scale for the PPI network, we propose a novel algorithm based the motif “thinking globally and fit locally” in machine learning community [36], [40]. By “thinking globally”, the structure of protein-protein network is globally revealed by the GGA at different scales. We will answer how to choose the optimal scale based on the “fitting locally”, which indicates that the globally discovered geometric structure should be able to match with local prior knowledge about the PPI in the network. We derive the fitting algorithm based on a standard metric, AUC statistic, for quantifying the capability of identifying members in different groups (PPI and NPPI in this work). The AUC, defined as the area under the receiver operating characteristic (ROC) curve, often interpreted as the probability that a randomly chosen missing connected pair of nodes (true positive) is given a higher score by GGA than a randomly chosen unconnected pair of nodes (true negative). The higher the AUC, the better the revealed global structure fits the original PPI network. We will choose the scale at which the AUC is maximized.
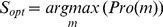
(7)where 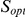 denotes the optimal propagation step, the 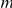 is the parameter of propagation step and 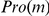 denotes the AUC at the step of 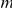. Note that the Equation 7 is not a close form definition but the optimal value can be found at a small step based on our experimental observations. As observed in **Figure 3**, probability value (AUC) increases initially and monotonically decreases after obtaining its maximum at step 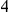. We then set the optimal propagation, 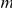, for the PPI network at 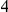.

### Optimal Local Fitting algorithm (OLF) for PPI assignment

The assumption of the correctness of geometry based approaches in analysis of PPI network is that a pair of proteins will be assigned an interaction if they are close in an embedded space whereas NPPIs correspond to points that are further away in that space [1], [22]. However, there is one question remaining to answer: how to determine an optimal threshold in the embedded space to judge whether two proteins are close enough in the space to properly assign them an interaction. In this paper, we proposed an approach based on optimally fitting the GGA matrix to local original PPI network.

In the original PPI network, we denote 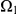 as the set of PPIs and 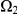 as the set of NPPIs. Given a threshold 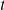, we could build a binary matrix in which the new interaction will be assigned if the geometric affinity is larger than the threshold, vice versa. We denote 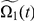 as the set of PPIs and 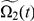 as the set of NPPIs in the newly determined PPI network. Based on these definitions, we define the following functions for each threshold 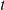:

True positive (TP) function
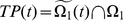
(8)
False positive (FP) function
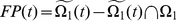
(9)
True negative (TN) function
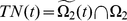
(10)
False negative (FN) function
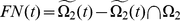
(11)
Match function
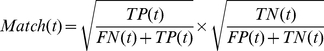
(12)

Given a threshold, the TP function measures the intersection between the new assigned PPIs set and the ground truth PPIs set, FP denotes the assigned edges which are not in the set of ground truth, TN denotes the intersection of new assigned NPPIs and ground truth of PPNs, and FN denotes new assigned PPIs in the ground truth of PPIs set. The match function evaluates how well the new assigned PPI network match with original local PPI network. From the mathematical point of view, we are about to solve the following optimization problem to find the optimal threshold value.
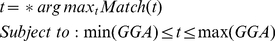
(13)


**Figure 4:**
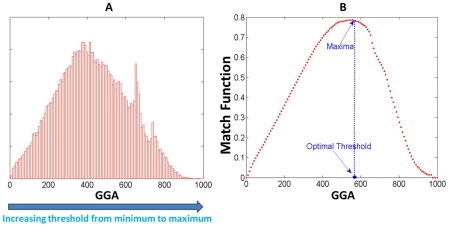
Determination of the threshold for PPI assignment by OLF. This figure illustrates the procedure of computing optimal threshold for PPI assignment. Figure (A) displays the histogram of global geometric affinity (GGA). Figure (B) displays the value of match function at different threshold. The optimal threshold for GGA corresponds the one with the maxima of match function.

The algorithm for solving the optimization problem is outlined below and **Figure 4** illustrates the process of finding an optimal threshold and assigning a new PPI network:

1. We vary the threshold from minimum to maximum found in GGA matrix among all pairs of proteins.
2. For a given threshold t, we compute true positive (TP) function, true negative (TN) function, false positive (FP) function and false negative (FN) function.
3. Based on the values obtained from the previous step, we compute match function.
4. The optimal threshold is the one with maximum match function value (see **Figure 4(B)**). To assign the new PPI network, we apply the optimal threshold to GGA matrix. The new PPI will be assigned if the geometric affinity is larger than the threshold, vice versa.

In **Figure 4(B)**, we can see that the match function starts zero, then increase until the peak value and then goes down to zero while varying the threshold from minimum to maximum of GGA. We can explain and understand the trend in the following way. When the threshold is equal to minimum value of GGA, all of the pairs of protein are assigned to the PPI, therefore the TN (min (GGA)) = 0 and then the Match (min (GGA)) = 0. When the threshold equal to maximum value of GGA, all of the pairs of proteins are assigned to NPPIs, therefore the TP (max (GGA)) = 0 and then the Match (max (GGA)) = 0. Although we do not prove the unique maxima value for match function in the current work, we observe the phenomenon by performing the experiment (see **Figure 4(B)**).

## Results

Based on the experimental setting in [1], [26], we provide the experiments to evaluate the performance of our method (denoted as GGA-method henceforth) on yeast PPI networks below. We use the receiver operating characteristic (ROC) curve, a graphical plot of the sensitivity versus (1 - specificity) and area under curve (AUC) to evaluate the performance of GGA-method. We present three experiments to assess the performance: 1) the evaluation by ROC curve and precision recall (PR) curve, 2) the evaluation by AUC at different noise levels, 3) the evaluation of PPI prediction by OLF algorithm.

### Data sources

We verify our approach on a publicly available and a high confidence S. cerevisiae network [41]. It consists of 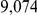 interactions amongst 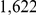 proteins (we denote it as CS hereafter). We use S. cerevisiae PPI network described by von Mering et al. [11] which contains 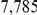 interactions among 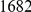 proteins (henceforth network is denoted by VM) and a network published in [42], which consists of 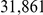 interactions amongst 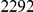 proteins (we denote it as Y2 hereafter).

### Experiment 1: Evaluations by ROC and PR Curve

The CS PPI network is used in this experiment. We compare GGA-method to MDS-method in the experiment in terms of the performance for differentiating the PPI and NPPI in the network. As MDS-method only works on well-connected component of a network, we take the largest connected component of CS network, which has 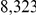 interactions between 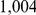 proteins. To validate the performance of GGA-method for differentiating the PPI and NPPI, we use the ROC curve and PR curve as the criteria. Both curves reflect how well GGA-method can differentiate the PPI from the NPPI based on the revealed relationship among proteins in the network. To plot the ROC curve and PR curve, we first define true positive (TP), false positive (FP), true negative (TN) and false negative (FN). The TP measures the intersection between the new assigned PPIs set and the ground truth PPIs set, FP denotes the assigned edges which are not in the set of ground truth PPIs set, TN denotes the intersection of new assigned NPPIs and ground truth of NPPIs, and FN denotes new assigned NPPIs which are not in the set of ground truth NPPIs. To compare with MDS-method, we choose low confidence edges in CS as ground truth PPI, which is the same ground truth used in [1]. The ROC and PR curves are computed based on GGA matrix as follows.

1. We vary the threshold from minimum to maximum value in the GGA matrix among all pairs of proteins.
2. For a given threshold, we compute TP, TN, FP and FN.
3. We compute the sensitivity rate (TP/(TP+FN)) and specificity rate (TN/(TN+FP)), precise (TP/(TP+FP)) and recall (TP/(TP+FN)). To plot the ROC curve, the horizontal axis represents (1 - specificity), and the vertical axis represents sensitivity. To plot the PR curve, the horizontal axis represents recall, and the vertical axis represents precision.

**Figure 5:**
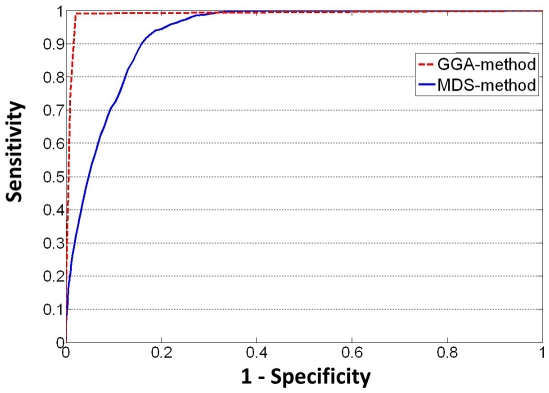
Comparison GGA-method and MDS-method by ROC curve. This figure plots the ROC curve for the comparison between GGA-method and MDS-method using the CS PPI network data. The vertical axis denotes the sensitivity, and the horizontal axis denotes 1-specificity.

The ROC curves are shown in the **Figure 5**. The illustrated results are encouraging in terms of the classfication performance. As we can see from the **Figure 5**, both GGA-method and MDS-method perform well on the clean data. However, the ROC curve of GGA-method is clearly above the curve for MDS-method. We can find that both specificity and sensitivity of GGA-method are over 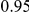 in this test, indicating an appealing performance in differentiating positive and negative PPI in network.

**Figure 6:**
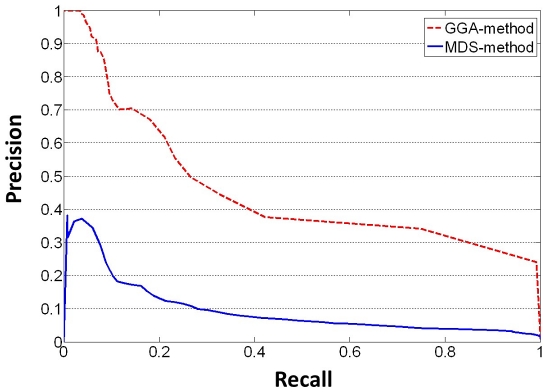
Comparison GGA-method and MDS-method by PR curve. This Figure plots the PR curve for the comparison between GGA-method and MDS-method using the CS PPI network data. The vertical axis denotes the precision, and the horizontal axis denotes recall.

Following the experimental setting in [1], we further plot the PR curve for accuracy analysis. The experimental result is shown in **Figure 6**. Because the PPI network is really sparse, the fraction of true PPI is orderly lower than the fraction of true NPPI. A random predictor would give less than 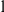 correct TP in 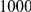 predictions, while the precision of PPI prediction of GGA-method can be over 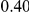 at a recall about 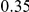 for the original PPI network. The precision and recall analysis in [1] provides a precision about 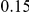 at a recall about 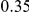. With their level of precision and recall, they are able to reveal at least twice as many PPI available in BioGRID. Our method is expected to give a much higher prediction of true PPI.

### Experiment 2: Evaluations by AUC at different noise levels

In this experiment, we demonstrate the performance of GGA-method in prediction of missing PPI and identification of spurious PPI in the noisy network. For an incomplete observed PPI network, we determine GGA for each pair of proteins in the network. We are interested in the pairs of proteins that have high GGA but are not connected in the observed network, and the pairs of proteins that have low GGA but are connected in the observed network. The first type of pairs of proteins are most likely candidates for missing PPIs, and the second type of pairs are most likely candidates for spurious PPIs. Our method is compared to MDS-methods in [1] in each test. For each network, we randomly remove a subset of connections for the simulation of missing PPI, and randomly insert a subset of connections for the simulation of spurious PPI. We attempt to predict the missing PPI and identify the spurious PPI. A well-established criteria for quantifying the performance of prediction algorithms in machine learning area is the AUC, which can be calculated by the area under ROC curve. The AUC is often interpreted as the probability that a randomly chosen missing connected pair of nodes (true positive) is given a higher score by GGA than a randomly chosen unconnected pair of nodes (true negative) [26]. A random predictor will give AUC of score 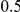, and the extent to which the AUC exceeds 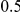 reflects how our prediction is better than chance.

We assess the performance of our method from two perspectives according to the tests on three PPI networks (CS,Y2,VM). First, we want to compare the performance in identification of spurious PPI using our method with that of MDS-method. We evaluate the comparison by gradually increase the insertions of the false PPI and attempt to identify those links using the topology information remaining in the network. Second, we want to compare the performance in predictions of missing PPI using our method with that of MDS-method. We evaluate the comparison by gradually increase the deletions of the true PPI and attempt to predict them using the topology information remaining in the network. The comparison result is displayed in the Table 1 and Table 2. The ‘Ins Ratio’ represents the ratio between the number of the inserted PPIs and the number of the true PPIs in the network. The ‘Del Ratio’ represents the ratio between the number of the deleted PPIs and the number of the true PPIs in the network. In Table 1, we can see that the AUC gradually decreases with the increase in the ratio of insertion from 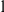 to 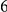. The comparison shows that GGA-method consistently outperforms MDS-method in identification of the spurious PPIs indicated by the higher values of AUC. Note that there is a certain ratio of deletion (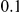) of PPIs applied across the tests. In Table 2, we can see that the AUC gradually decreases with the increase in the ratio of deletion from 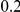 to 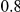. The comparison of two methods shows that GGA-method is consistently better than that of MDS-method in predicting missing PPIs indicated by the higher values of AUC. Note that there is a certain ratio of insertion (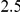) of PPIs applied across the tests. From the table, we can observe that both methods are more resistant to insertion noise (spurious PPI) than to deletion noise (missing PPI). This is because the number of the NPPIs is orderly larger than the number of the PPIs. A large fraction of deleted PPIs leads to dramatic deterioration of intrinsic geometric structure of network. We also notice that GGA-method is able to perform reasonably even when 80% of true PPIs are deleted or 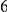 times of false PPIs are inserted. However, at this level of noise, the performance of MDS-method is close to a random predictor, indicated by the AUC value (near 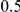). This test confirms that GGA-method is robust against two types of noise.

**Table 1:** Comparison of AUC test of GGA and MDS methods by insertion noise.

	Ins Ratio	1	2	4	6
CS	GGA-method	0.9538	0.9375	0.8964	0.8583
	MDS-method	0.7106	0.7021	0.4793	0.4580
Y2	GGA-method	0.8470	0.8013	0.7743	0.7482
	MDS-method	0.7100	0.6040	0.4276	0.4196
VM	GGA-method	0.9480	0.8956	0.8636	0.8347
	MDS-method	0.7099	0.6807	0.6913	0.6137

AUC values are computed for both methods under certain level of insertions of false positive PPIs in the original network. ‘Ins Ratio’ indicates the ratio between inserted false PPIs and the number of PPIs in the original network and corresponding AUC values are filled in the table.

**Table 2:** Comparison of AUC test of GGA and MDS methods by deletion noise.

	Del Ratio	0.2	0.4	0.6	0.8
CS	GGA-method	0.9307	0.9114	0.8661	0.7131
	MDS-method	0.7059	0.6975	0.6161	0.5226
Y2	GGA-method	0.7922	0.7676	0.7277	0.6608
	MDS-method	0.5413	0.5807	0.5973	0.5666
VM	GGA-method	0.8912	0.8673	0.7893	0.7143
	MDS-method	0.6602	0.6023	0.5091	0.3635

AUC values are computed for both methods under certain level of deletions of true positive PPIs in the original network. ‘Del Ratio’ indicates the ratio between removed true PPIs and the number of PPIs in the original network and the corresponding AUC values are filled in the table.

We explain why MDS-method performs worse than GGA-method against insertion and deletion noise. The metric revealed by MDS is based on the shortest path traveled from one protein to another protein in the network [1]. Before the deletion of the PPI, the shortest path between the pair of proteins is 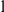 as they are linked. However, the length of the shortest path increases if PPI no longer exists between them. Before the insertion of the PPI, the shortest path between the pair of proteins is larger than 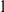 as they are not directly linked. The path-length might be a very large if two nodes are really far away. However, the shortest path would be changed to 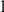 if a link is introduced between them because of noise. The short-circuit noise is a typical topological noise in computational geometry area [43] which is usually overcame by the global geometric metric, for example the graph Laplacian based representation. While GGA-method evaluates the global affinity of two nodes taking into account all existing connections, therefore, they are more robust to both missing PPI and spurious PPI according to the results in the table.

To demonstrate the computation efficiency of GGA-method, we compare the computation time for both methods. The Table 3 contains the comparison result. We carried out 10 experiments and recorded the computation time for both methods. It can be found that GGA-method runs much faster than MDS-method (nearly 40 times faster). The speed of the GGA-method over the MDS-method arises from its the eigen-decomposition-free advantage. This is even more obvious if the size of the PPI network is huge and its adjacency matrix is singular in some cases.

**Table 3:** Comparison of GGA-method and MDS-method in computation time.

Method	E1	E2	E3	E4	E5	E6	E7	E8	E9	E10
GGA-method	6.21	6.36	7.01	6.37	7.51	6.73	6.53	6.7	6.28	6.65
MDS-method	260.18	258.75	263.85	260.76	264.96	247.4	252.71	255.29	271.57	273.56

Note that the unit used for measuring computation time is second, E denotes experiment, for example, E1, means the first experiment.

### Experiment 3: Evaluations of PPI prediction by OLF algorithm

The Optimal Local Fitting algorithm (OLF) is used to determine the threshold for the PPI assignment from the GGA matrix. The test result is shown in the Table 4. We assess the performance by the value of true positive accuracy (TPA). The TPA is the percentage of the recovered PPIs out of the ground truth PPI, which is defined as TP/(TP+FN). Two true positive accuracy, TPA1 and TPA2, are evaluated for each method with different ground truth PPI. TPA1 is the percentage of the recovered PPIs out of the deleted PPI in the ground truth, and TPA2 is the percentage of the recovered PPIs out of all of ground truth PPI. When the number of the insertions is zero, the MDS-method has computational difficulty because the network is not well-connected especially after a certain amount of PPIs is deleted, which does not satisfy the conditions of PPI network required by the MDS-method. In addition, the original MDS-method manually choose the threshold for the PPI assignment, for example, 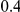 in the second experiment presented in [1]. The strategy for the manual selection of the threshold is not well explained in their work. Based on our test, the MDS-method performs unstably when the threshold is manually set 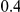 as used in [1]. Therefore, we use our OLF algorithm for the determination of the threshold for MDS-method in this test, which greatly improves the performance of MDS-method in both stability and accuracy. We carry out the following two tests:

1. In the first test, we randomly insert the a certain amount of PPIs (Ins Ratio is 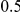) to the network and delete various amounts of PPIs (Del Ratios are 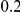, 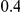 and 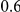).
2. In the first test, we randomly insert the a certain amount of PPIs (Ins Ratio is 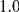) to the network and delete various amounts of PPIs (Del Ratios are 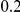, 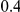 and 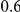).

**Table 4:** Comparison of GGA and MDS method in prediction performance by OLF algorithm.

	CS				Y2				VM			
	GGA-method		MDS-method		GGA-method		MDS-method		GGA-method		MDS-method	
	TPA1	TPA2	TPA1	TPA2	TPA1	TPA2	TPA1	TPA2	TPA1	TPA2	TPA1	TPA2
1	0.9449	0.9517	0.7767	0.8216	0.8387	0.8906	0.6775	0.7173	0.8735	0.9119	0.7495	0.8311
2	0.9234	0.9339	0.7484	0.8124	0.8091	0.8542	0.6219	0.6526	0.8513	0.8798	0.7026	0.7920
3	0.8778	0.8906	37605	0.8057	0.7785	0.8062	0.5322	0.5542	0.8182	0.8450	0.6836	0.7571
4	0.9366	0.9508	0.7574	0.7953	0.8316	0.8611	0.6533	0.6912	0.8703	0.9048	0.7174	0.8054
5	0.9151	0.9315	0.7520	0.7978	0.8088	0.8359	0.5444	0.5790	0.8504	0.8741	0.7036	0.7753
6	0.8738	0.8819	0.7419	0.7897	0.7576	0.7789	0.5568	0.5926	0.8114	0.8310	0.6637	0.7430

We analyze the results in Table 4 for two tests separately.

1. The result for the first test is presented in the first three rows (labeled 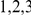 in the first column). In the table, we can find that both TPA1 and TPA2 are around 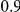 by GGA-method, suggesting most of the deleted PPIs are correctly recovered. The value of TPA is around 0.88 even with the 60% of true PPI is deleted. In contrast, the values of TPA for MDS-method are around 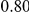 in the test. Some of the TPA values of MDS-method, for example, the result in Y2 test, are nearly 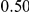, while GGA-method is able to remain around 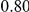 in the same test.
2. The result for the second test is presented in the second three rows (labeled 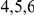 in the first column). It indicates that GGA-method is insensitive to the insertion noise because the performance of recovering the missing PPIs is not affected by introducing a higher number of insertions of PPIs (the insertion ratio increases from 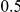 to 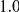). The comparison of the performance between two methods is obvious according to the TPA values in the table.

## Discussion

The limitations of the current high-throughput measurements techniques inherently give rise to a large amount of spurious and missing PPIs. To clean the network, people often try to integrate multiple data sources, such as gene expression arrays and proteomics to improve the quality of PPIs in a network. Recently, geometric based approaches, which are only based on the topology of the PPI network, are very promising as those approaches are independent from other prior knowledge except for topology of the PPI network. However, the large amount of noisy PPIs poses a great challenge to the geometric based computational approaches. Robust geometric structural understanding methods are the prerequisite for capturing the intrinsic geometric structure which is hidden behind the noisy PPI network data.

### A robust global geometric metric for noisy and incomplete PPI network

Biological data, like the PPI data, are often observed in an incomplete manner with high noise. Any method, if simply based on the metric of a small number of local PPI network, is likely to be overwhelmed by the noise and incompleteness. It is of great importance to place the data in a statistical model and take into account all the pieces of local information simultaneously, in order to generate the knowledge behind the overall global structure of the data. Globally consistent metric, like the global geometric affinity proposed in this work, measure the relationship considering the optimal arrangement of all the data samples. Therefore, even if the local incomplete and noisy pairs of PPIs are not able to reveal the internal global structure, given sufficient samples of PPIs and considering the entire set of pair-wise linkages simultaneously, our GGA-method is able to reveal the intrinsic metric hidden in the very noisy and incomplete PPI data. The excellent robustness against noise is highlighted by its good performance at a large number of insertions and deletions introduced.

### Minimizing the use of prior knowledge

Biological experimental measurements are usually time consuming and costly. The computational approaches are proposed to benefit biological experiments and to better characterize network data sets by minimizing the use of the prior knowledge from biological experiments. In recent years, the geometric features of PPI network have been proven to provide new insights into the function of individual proteins, protein complexes and cellular machinery as a complex system. These approaches take advantage of the geometric characteristics behind the PPI network, which enables to evaluate the relationship among the protein-protein interactions and analyze the network characteristics. These approaches based only on the geometric topology formed by connections among proteins and has been recently developed to de-noise the observed PPIs network. The common hypothesis for these methods is that the existence of the geometric structure for PPI networks and the topology knowledge is crucial in determining the PPIs in the network [22], [23]. Under this assumption, a pair of proteins will be assigned an interaction if they are close in an embedded space whereas protein protein non-interactions correspond to points that are further away in that space [1].

### Algorithm efficiency

The efficiency of a computational approach for most biological problems is vital in real applications due to the high-throughput nature of the data. The existing geometry based methods for the de-noising the PPI network, for example, MDS-method, are computationally expensive and even intractable for large and incomplete PPI networks. This is because these methods include the eigen-decomposition to compute the explicit embedding coordinates and then compute the global geometric distance. In our method, we completely decouple the global metric from the eigen-decomposition problem by proposing the GGA. The eigen-decomposition-free method give our method a distinct advantage that we can totally get rid of the issues caused by the eigendecomposition. Therefore, our proposed method is able to apply on the sparse and huge size PPI network and finish the de-noising in an efficient way. These virtues account for the superiority of our proposed in the real applications for de-noising PPI networks.

### Limitations and future directions

This paper presents our first implementation, with very promising results in the completed tests. However, the noise properties in raw PPI data can be different from the simulated random deletions and insertions used in existing tests. The performance of our method and its general applicability in de-noising a PPI network generally confirm the robustness of our methods but still need further work to improve by testing more real challenging PPI data. The parameter of propagation step plays a critical role in looking through the geometric structure from multiple scales. Although we provide a probability based algorithm to determine the optimal parameter, we have not given a rigorous proof. In our future work, we will come up a good strategy based on this parameter to investigate the raw PPI network at different level of details. The OLF algorithm is proposed to numerically determine the optimal threshold without a closed form solution or proof to that optimization problem. Furthermore, GGA-method is a general method and applicable to a wide range of problem domains, for example, the reconstruction of the air transportation network.
